# *Salmonella* serovars in a tertiary hospital in Turkey, 2015-2017: molecular epidemiology, antimicrobial resistance and molecular characteristics of resistance mechanisms

**DOI:** 10.4314/ahs.v25i2.10

**Published:** 2025-06

**Authors:** Tugce Unalan-Altintop, Selay Demirci-Duarte, Ozgen Koseoglu-Eser, Aycan Gundogdu, Aslı Cakar, Revasiye Gulesen, Belkıs Levent, Belgin Altun, Banu Sancak, Deniz Gür

**Affiliations:** 1 Hacettepe University, Faculty of Medicine, Department of Medical Microbiology, Ankara, Turkey; 2 Erciyes University, Faculty of Medicine Department of Medical Microbiology, Kayseri, Turkey; 3 Genome and Stem Cell Center (GenKok), Erciyes University, Kayseri, Turkey; 4 Turkish Public Health Agency, Department of Microbiology Reference Laboratories, Ankara, Turkey; 5 Hacettepe University, Vocational School of Health Services, Ankara, Turkey

**Keywords:** Salmonella, colistin, trimethoprim-sulfamethoxazole, ESBL, ciprofloxacin, PFGE

## Abstract

**Background:**

Increase in antimicrobial resistance poses a global threat for treatment of Salmonellosis.

**Objectives:**

In this study, serovar distribution, antimicrobial susceptibility, resistance genes, and clonal diversity were characterized in clinical *Salmonella* isolates.

**Methods:**

In this study, serovar distribution of clinical *Salmonella* isolates was characterized by the Kauffman-White scheme. The antimicrobial susceptibility was determined by the broth microdilution method. Resistance genes and clonal diversity were investigated by PCR and PFGE.

**Results:**

The serovars were *Salmonella enterica* serovar Enteritidis (n=92), *Salmonella enterica* serovar Typhimurium (n=14), and others (n=25). Resistance to ciprofloxacin, ceftriaxone, trimethoprim-sulfamethoxazole, azithromycin, meropenem, and colistin were 13.3%, 2.2%, 5.2%, 3.0%, 0%, 29.6%, respectively. Two ESBL-positive isolates carried TEM-type β-lactamases. Carbapenemases, *mcr*-1 and *mcr*-2 could not be detected. Colistin-resistant S. Enteritidis isolates were grouped in 4 pulsotypes [A1 (n=12), A2 (n=2), A3 (n=13) and B (n=1)]. All except one (B) were found closely related.

**Conclusions:**

A relative decrease in resistance to trimethoprim-sulfamethoxazole was detected with time. Trimethoprim-sulfamethoxazole and azithromycin can be good alternatives to the widely-used ciprofloxacin and third-generation cephalosporins. High resistance of colistin and ciprofloxacin may be due to the extensive use of antibiotics in poultry, which highlights the significance of one health concept.

## Introduction

Non-typhoidal *Salmonella* (NTS) infections generally present as self-limiting diarrhea; however, severe and invasive forms can occur in the elderly, children and patients with underlying co-morbidities^1^. A non-specific fever, bacteremia, endovascular infections, osteomyelitis or abscesses are all symptoms of severe infections and can lead to a high number of deaths every year especially in sub-Saharan region^2^. For these infections, ciprofloxacin, trimethoprim-sulfamethoxazole (TMP-SMX), and/or ceftriaxone are used in the treatment which may cause the emergence of resistance. Foodborne zoonotic transmission, contact with animals, and exposure to the environment are all possible ways for humans to acquire antimicrobial resistance^3^.

*Salmonella* Enteritidis and *Salmonella* Typhimurium were the most common serovars isolated from many vertebrates worldwide whereas other isolates were associated with invasive diseases include Dublin, Choleraesuis, Virchow, Infantis, Newport, and Heidelberg^4^. S. Infantis infections are increasing and linked to chickens; while S. Typhimurium infections are decreasing, which may be due to poultry vaccination^5^-^8^.

In the Centers for Disease Control (CDC)'s Antibiotic Threats in United States Report, 2019, drug-resistant NTS infections are grouped within the serious public health threats, and the resistance rate to at least one essential antibiotic rate is increasing^9^. Resistance can occur by two different mechanisms either chromosomal or plasmid-mediated^10^. The horizontal gene transfer in *Salmonella* species is an important mechanism in the multi-drug resistance development^10^. It is critical to reduce the dissemination of NTS infections, by effective surveillance as well as tracking the routes of acquired horizontal resistance.

In systemic infections due to *Salmonella* isolates, resistance to fluoroquinolones and production of extended spectrum beta-lactamases (ESBLs) are reported with increasing frequency^10^. Due to the worldwide distribution of antibiotic resistance in Salmonella infections, a need for the use of antimicrobials with extended spectrum of activity has emerged. Frequent use of antibiotics in poultry for the purpose of mainly growth promotion and prophylaxis has resulted in plasmid-mediated colistin resistance^11^.

In this study, serovar distribution, antimicrobial susceptibility, antimicrobial resistance genes and clonal diversity were characterized in clinical *Salmonella* isolates from a reference tertiary care center in Turkey. The aim of the study is to monitor the resistance in clinical *Salmonella* isolates, to investigate the mechanism behind the resistance and the clonal relationship of the resistant isolates. Also, our aim is to demonstrate the change in resistance rates over time in our center.

## Materials and methods

### Collection and identification of the isolates

A total of 131 clinical *Salmonella* isolates were collected consecutively in Hacettepe University Clinical Microbiology Laboratories during 2015-2017. The isolates were identified by matrix-assisted laser desorption ionization time of flight mass spectrometry (MALDI-TOF MS) (bioMerieux, France). The isolates were serotyped following the White–Kauffmann–Le Minor scheme by slide agglutination with O and H antigen-specific antisera (Statens Serum Institute, Denmark)^12^.

### Antimicrobial susceptibility testing

In vitro susceptibility testing of the isolates was performed using the broth microdilution method. Minimum inhibitory concentrations (MICs) of ciprofloxacin, ceftriaxone, TMP/SMX, azithromycin, meropenem, and colistin (Sigma Aldrich Corp., USA) were determined and interpreted according to EUCAST v8.1 guideline^13^. *Escherichia coli* ATCC 25922, *E. coli* NCTC 13846 (for colistin) and *Staphylococcus aureus* ATCC 25923 (for azithromycin) were included as quality controls.

### DNA Extraction

DNA of the isolates was extracted by the boiling lysis method. Briefly, bacterial strains were inoculated to Columbia Agar with 5% sheep blood (Becton Dickinson, Germany) and incubated overnight at 37 °C. A loopful bacteria was suspended in sterile distilled water and boiled for 10 minutes. After boiling, the tube was centrifugated at 12000 rpm for 5 minutes. The supernatant was transferred to a sterile microcentrifugation tube and stored at -20 °C^14^.

### Determination of antimicrobial resistance determinants

ESBL production was investigated phenotypically with combined disk diffusion method (ROSCO Diagnostics, Denmark). All ceftriaxone non-susceptible isolates were further tested for the presence of four frequent ESBL-types encoding TEM-, SHV-, OXA-, and CTX-M-type beta-lactamases by PCR using primers based on previous literature^15^-^17^. Isolates with high MICs (MIC ≥0.125 mg/L) for meropenem were also investigated for the presence of OXA-48, IMP-, VIM-, NDM-, SPM-, AIM-, and KPC-type carbapenemases as previously described^18^. The presence of plasmid-mediated colistin resistance genes (mcr-I and mcr-II) were investigated in isolates with high MICs (≥2mg/L) for colistin by PCR using primers previously described^19^,^20^. All amplicons were sequenced and compared with reference strains in NCBI GenBank records.

### Molecular epidemiologic analysis

The clonal relationship of colistin-resistant S. Enteritidis isolates was investigated with pulsed-field gel electrophoresis (PFGE). PFGE was performed according to the PulseNet standardized protocol with XbaI digestion enzyme^21^. Banding patterns were photographed under UV transillumination and analyzed using Chemidoc MP Imaging System and software (Biorad/USA). A 2% difference in band position was determined to develop a dendrogram. The percent similarity of the banding patterns was estimated using Dice coefficient. Each unique electrophoretic band pattern was interpreted as a pulsotype^22^.

## Results

### Clinical and demographic features of patients

A total of 131 *Salmonella* isolates were included in the study which were collected consecutively per patient in Hacettepe University Clinical Microbiology Laboratories during 2015-2017. The type of samples investigated were as follows: stool (n=117), urine (n=5), blood (n=5), tissue (n=2), peritoneal fluid (n=1), pus (n=1). Among the patients, 52 (39.7%) of them were female and 95 (72.5%) were children. In samples, 20 (15.3%) of them were from hospitalized patients, and others from outpatient clinics. Hospitalized patients were from pediatrics (n=4), oncology (n=4), general medicine (n=3), hematology (n=2), pediatric oncology (n=1), intensive care unit (n=1), infectious diseases (n=1), nephrology (n=1), general surgery (n=1), obstetrics and gynecology (n=1), and orthopedics (n=1).

### *Salmonella* serotypes

A total of 14 serovars belonging to five serogroups were identified among the 131 isolates (Table 1).

**Table 1:** Salmonella serogroups and serovars detected by Kauffman-White Scheme

Serogroups	Serovars	n	Total
O:4 (B)			20
	Paratyphi B	4	
	Saintpaul	2	
	Typhimurium	14	
O:7 (C1)			7
	Augustenburg	1	
	Infantis	4	
	Mbandaka	1	
	Virchow	1	
O:8 (C2-C3)			11
	Corvallis	1	
	Hadar	3	
	Kentucky	4	
	Muenchen	2	
	Newport	1	
O:9 (D1)			92
	Enteritidis	92	
O:3,10 (E1)			1
	Anatum	1	

### Antimicrobial susceptibility and resistance determinants

The MICs determined by broth microdilution method are shown in Table 2.

**Table 2:** Antimicrobial resistance profiles of Salmonella isolates

Antibiotics	Range (mg/L)	MIC_50_ (mg/L)	MIC_90_ (mg/L)	Resistance n (%)
Ciprofloxacin	≤0.01 - 8	≤0.01	0.125	18 (13.3)
Ceftriaxone	≤0.01 ->32	0.03	0.125	3 (2.2)
TMP/SMX	≤0.01 - 8	0.125	2	7 (5.2)
Azithromycin	0.125 ->256	8	16	4 (3.0)
Meropenem	≤0.01 ->32	0.01	0.03	0 (0.0)
Colistin	≤0.01 ->32	2	8	40 (29.6)

Of the three ceftriaxone-resistant isolates, two were identified as S. Enteriditis and one as S. Infantis. All were isolated from stool cultures. Two ceftriaxone-resistant isolates were determined as ESBL positive by combined-disc method and confirmed to be producing TEM-type beta lactamases by PCR. Both were resistant to ciprofloxacin and colistin, but susceptible to TMP/SMX and meropenem.

None of the isolates were resistant to meropenem. None of the isolates with MICs higher than 0.125 mg/L were positive for the carbapenemase genes.

Eighteen of the patients were found to harbor low level resistance to ciprofloxacin. The serovar distribution were as follows: Enteritidis (n=10); Infantis (n=3); Kentucky (n=3); Mbandaka (n=1); Virchow (n=1). All isolates except one were isolated from stool; one was from a blood culture.

Seven of the isolates were resistant to TMP/SMX. Three of them were also non-susceptible to ciprofloxacin but susceptible to ceftriaxone. The serovar distribution were as follows: Enteritidis (n=3); Infantis (n=1); Kentucky (n=1); Mbandaka (n=1); Typhimurium (n=1).

Four isolates (S. Typhimurium, S. Kentucky, S. Anatum and S. Corvallis) were resistant to azithromycin.

Forty of the isolates were resistant to colistin. Twenty-eight of them were identified as S. Enteritidis. Other colistin resistant non-Enteritidis *Salmonella* isolates were: S. Typhimurium (n=3), S. Infantis (n=2), S. Paratyphi B (n=2), S. Corvallis (n=1), S. Hadar (n=2), S. Augustenburg (n=1), S. Anatum (n=1). None of the isolates harbored mcr-1 or mcr-2. Thirty-four of the isolates were from stool cultures, five from urine and one from tissue.

### PFGE analysis

**Figure 1:**
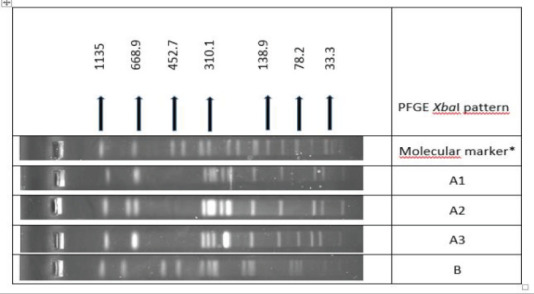
PFGE profiles (XbaI) of colistin resistant S. Enteritidis isolates

Colistin-resistant S. Enteritidis isolates were grouped in 4 pulsotypes [A1 (n=12), A2 (n=2), A3 (n=13) and B (n=1) with XbaI enzyme. All isolates except one (B) were found closely related. A1 and A2 patterns differed by one band, A1 and A3 patterns by two bands, A2 and A3 patterns by three bands (Figure 1).

*Molecular marker (S. Braenderup; H9812)

## Discussion

NTS infections are common but a significant public health problem worldwide. In Turkey, the most common *Salmonella* serovars were S. Enteritidis and S. Typhimurium in a multicenter study, concordant with our study^23^. Antimicrobial resistance in NTS is considered as a serious threat by CDC^24^. Ciprofloxacin, azithromycin, ceftriaxone, ampicillin, or trimethoprim-sulfamethoxazole were characterized as essential antibiotics in the treatment of salmonellosis by CDC^24^. According to National Antimicrobial Resistance Monitoring System of CDC in 2017, the rate of resistance to three or more essential antibiotics was 2% in human isolates in USA^24^. In our study, two of the isolates were resistant to both ciprofloxacin and ceftriaxone, and three were resistant to ciprofloxacin and TMP/SMX, but none was resistant to three essential antimicrobials.

Low level resistance to ciprofloxacin is a greater concern since it is the first line choice for invasive and localized gastrointestinal infections in adults. Plasmid-mediated resistance causes challenges in the treatment of *Salmonella* epidemics. In Europe, according to results of European Food Safety Authority, Antimicrobial Resistance Report for *Salmonella* spp. isolated from citizens of EU Member States, Iceland, Norway and Switzerland, ciprofloxacin resistance was 13.5%^25^. In National Antimicrobial Resistance Monitoring System of CDC in 2017, ciprofloxacin resistance was reported as 7%^24^. In a previous study conducted in our center, resistance to ciprofloxacin was found to be 12.3% in NTS isolates in 2006^26^. Ciprofloxacin resistance is slightly increasing over time in our center, which we believe can be explained by the increase in misuse of this drug in the hospitals. Various studies demonstrate an increase in non-susceptibility to fluoroquinolones^24^,^27^-^30^. Fluoroquinolone-resistant NTSs are listed among resistant bacteria of international concern in the World Health Organization (WHO) report on the Global Response on Surveillance^31^. The increasing resistance highlights the need for effective antimicrobial stewardship strategies and fast and accurate monitoring of resistance to fluoroquinolones in *Salmonella* infections.

Among NTS, extended-spectrum cephalosporin resistance is a serious public health concern. In neonates, the first choice of treatment in meningitis and bacteremia is cefotaxime and extended spectrum cephalosporins are widely favored in older children. In Europe, cefotaxime resistance was reported to be 1.8% in NTS isolates from humans collected by EFSA^25^. According to National Antimicrobial Resistance Monitoring System of CDC in 2017, ceftriaxone resistance was reported as 3% in human isolates in USA^24^. ESBL genes are emerging worldwide and certain serovars are found to be more likely to carry some plasmids or resistance-encoding genetic elements^32^,^33^. ESBL genes are found on plasmids, which can be easily transported between and among bacteria. Certain ESBL genes are derived from mutant forms of well-known plasmid-mediated β-lactamases, such asTEM/SHV, while others are derived from bacteria found in the environment, like CTX-M^34^. In our study, three were resistant to ceftriaxone and two of these isolates harbored TEM-type beta lactamase (one S. Enteritidis and one S. Infantis). In a previous study from our center, two ESBL positive *Salmonella* isolates also harbored TEM-1 beta-lactamase accompanying SHV-2, SHV-2a and SHV-5a^35^. Another study from Turkey reported a clinical isolate of S. Virchow carrying CTX-M3^36^. Although cephalosporin resistance is still rare, given the high use of cephalosporins in children, ESBL production may be expected to increase and should be monitored carefully.

In multi-drug resistant *Salmonella* infections where third generation cephalosporins and ciprofloxacin resistance occur, carbapenems are the drugs of choice. Carbapenemase production in NTS is rare but has been reported previously^37^. In a recent study from Turkey, OXA-48 gene was found in a clinical S. Senftenberg isolate^38^. In our study, all of the isolates were susceptible to meropenem and none harbored carbapenemase genes. Future increases in resistance would make treating invasive *Salmonella* infections much more challenging.

TMP/SMX is a traditional drug in the treatment of Salmonellosis, but its use has been limited due to the increase in resistance over time. In our study, resistance to this drug was found as 5.2%. In a previous report from our center, resistance was reported as 7%^39^. A relative drop in the resistance was observed in TMP/SMX, which can be explained by the replacement of traditional drugs by ciprofloxacin and third generation cephalosporins. Several studies from Europe and Asia also reported a decrease in TMP/SMX resistance^11^,^27^. TMP/SMX is still a good therapeutic option given the resistance to third generation cephalosporins and ciprofloxacin is increasing.

Colistin is widely used against multidrug-resistant Enterobacterales and is frequently used in poultry worldwide^40^. In recent years, plasmid-mediated colistin resistance (mcr) genes have been widely documented in Enterobacterales including *Salmonella* isolates obtained from clinical cases, food, animal, or environmental sources from European countries and Asian countries, especially from China^41^.

In the majority of these nations, polymyxins were heavily used in the production of food animals 42. In a study, almost all breastmilk from mothers had veterinary drug residues including polymyxins in Turkey^43^

The use of polymyxins in poultry is of great concern, since plasmid-mediated colistin resistance presents a risk to the treatment of multiple drug resistant organisms. In general, S. Typhimurium is the most frequent serovar which harbors mcr genes^44^. However, in our study, among 40 colistin-resistant clinical isolates, only three of them were S. Typhimurium and S. Enteritidis was the most prevalent serovar (n=28). MIC50 value of colistin is found as 2 mg/L and MIC90 is 8 mg/L, showing that majority of the isolates has MIC values around the resistance breakpoint, hence 24 of the isolates had the MIC of 4 mg/L. Molecular epidemiological analysis of these isolates by PFGE demonstrated that these S. Enteritidis isolates were clonally related except one. Same clonal resistant isolates may have caused *Salmonella* outbreaks in humans probably spreading from the same source. This could be the reason for the high rate of colistin resistance in our study.

After the first detection of *mcr-1* genes, additional mcr genes from *mcr-2* to *mcr-9* were found^45^. There were concerns regarding the acquisition of these resistance genes from livestock and their emergence in clinical isolates^46^. In our study, 29.6% of the clinical *Salmonella* serovars were resistant to colistin. Most of these colistin resistant isolates were S. Enteritidis (70%) and they grouped in four pulsotypes which were closely related except one. However, among all these colistin resistant isolates none of them harbored *mcr-1* or *mcr-2* genes. There were no previous reports on colistin resistance in *Salmonella* isolates recovered from patients with salmonellosis; however, a recent study detected *mcr-1* gene in an *Escherichia coli* isolate from wastewater^47^. Since there are studies showing that *mcr* genes other than *mcr-1* and *mcr-2* were present in *Salmonella* species^48^, mechanism of colistin resistance should be further investigated for other plasmid mediated mcr types or chromosomally encoded resistance.

Azithromycin is a common drug used to treat cases of uncomplicated enteric fever with its good tissue penetration and concentration in reticuloendothelial cells. However, there is an emerging resistance in enteric fever cases as well as in non-typhoidal Salmonella cases 49. In some studies even outbreaks of azithromycin resistant non-typhoidal Salmonella were reported^50^. In National Antimicrobial Resistance Monitoring System of CDC in 2017, azithromycin resistance was reported as 0.5%^24^. In our study, only four isolates (3%) were resistant to azithromycin (MIC_90_ 16 mg/L). In Europe, azithromycin resistance was reported as 0.8%^25^. Azap et al. reported MIC_90_ value as >16mg/L^51^. In another study from Turkey, no isolates were found to be resistant to azithromycin, and MIC_90_ was 4 mg/L^38^. Even though azithromycin resistance seems low among our isolates, effective surveillance studies should be conducted with more non-typhoidal Salmonella isolates to tackle the concerning azithromycin resistance.

## Limitations

Firstly, we only investigated *mcr-1* and *mcr-2* genes in colistin resistant isolates; however, other *mcr-3, mcr-4, mcr-5* and *mcr-9* have also been detected in *Salmonella*^52^. We performed PFGE analysis only for colistin resistant S. Enteritidis isolates, while screening all the isolates may have provided a deeper understanding of the molecular epidemiology of *Salmonella* in our center. Also, investigation of resistance in animal-rooted *Salmonella* isolates and comparing the results with human isolates could have demonstrated the link of colistin use in animal husbandry to increased resistance in human.

Resistance to essential antibiotics in the treatment of Salmonellosis is a global public health problem and it should be monitored carefully. In our study, an increase in low level resistance to ciprofloxacin has been observed. The ESBL production was low and carbapenem resistance did not occur. There has been a relative decrease in TMP/SMX resistance in our center, offering TMP/SMX as a good alternative for treating *Salmonella* infections. Few azithromycin resistant isolates were detected. High rates of colistin and ciprofloxacin resistance may be given credit for the immense use of these drugs in poultry. The increasing resistance of *Salmonella* should be managed with one health perspective and excessive use of antibiotics in poultry should be limited.
